# COVID-19 in the U.S. during pre-vaccination period: Shifting impact of sociodemographic factors and air pollution

**DOI:** 10.3389/fepid.2022.927189

**Published:** 2022-10-26

**Authors:** Chaya Chaipitakporn, Prashant Athavale, Vijay Kumar, Thevasha Sathiyakumar, Marko Budišić, Shantanu Sur, Sumona Mondal

**Affiliations:** ^1^David D. Reh School of Business, Clarkson University, Potsdam, NY, United States; ^2^Department of Mathematics, Clarkson University, Potsdam, NY, United States; ^3^Department of Biology, Clarkson University, Potsdam, NY, United States

**Keywords:** COVID-19, infection, fatality, ethnicity, air pollutants, Hispanic, education

## Abstract

Pandemic “wave” usually refers to the rise and fall of the infections with time, however, for a large country, the variations due to geographical location could be considerable. In this work, we investigated COVID-19 infection and fatality across the U.S. during the pandemic waves in the pre-vaccination period (January 2020–December 2020). Focusing on counties with a population ≥100,000, the data from the entire period were first segmented into two equal phases roughly corresponding to the first pandemic wave and subsequent surge, and each phase was further divided into two zones based on infection rate. We studied the potential influences of six sociodemographic variables (population density, age, poverty, education, and percentage of Hispanic and African American population) and four air pollutants (PM_2.5_, NO_2_, SO_2_, and O_3_) on the differences in infection and fatality observed among different phases and zones. We noticed a distinct difference in the overall impact of COVID-19 between the two phases of the pre-vaccination period with a substantial decrease in the fatality in the second phase despite an increase in the infection. Analysis using log-linear regression modeling further revealed a shift in the impact of several risk factors considered in this study. For example, population density and lesser education were found to be significant for infection during the first phase of the pandemic alone. Furthermore, population density and lesser education along with poverty and NO_2_ level had a significant contribution to fatality during the first phase of the pandemic, while age over 65 years was important in both phases. Interestingly, the effects of many of these factors were found to be significant only in the zones with higher infection rates. Our findings indicate that the impacts of several well-known sociodemographic and environmental risk factors for COVID-19 are not constant throughout the course of the pandemic, and therefore, careful considerations should be made about their role when developing preventative and mitigative measures.

## Introduction

Recent research has shown that elevated levels of various air pollutants negatively affect the prognosis of COVID-19. Numerous researchers have also revealed a disproportionate impact of groups with lower socioeconomic levels in the U.S. The spread of infectious diseases is usually referred to as a wave in time, however, the spatial component of the spread can play an important part in the spread of a disease (1). The spatial component can not be ignored, especially in a large country such as the U.S. In this paper, we study the spatiotemporal nature in pre-vaccination period of the COVID-19 pandemic. We divide this period into two time-phases, and group the counties in two zones based on their infection rates. We attempt to understand the effects of various risk factors in the two Phases, and the two zones separately. This paper analyzes the effect of environmental, demographics, and geographical attributes on COVID-19 infection and fatality across the U.S. We also aim to quantify the differences in the first two phases of the pandemic.

### Sociodemographic factors affecting COVID-19

High volume of population in one place especially if it is dense, increases the air pollution. Indeed, Cole and Neumayer (2) observe that the high population leads to higher levels of SO_2_ emission. Moreover, Páez-Osuna et al. (3) find that higher population density leads to higher COVID-19 mortality. Hence, we consider population density as one of the factors in our analysis.

Moreover, other demographic factors such as age, race, and education play an essential part in the COVID-19 prognosis. Mueller et al. (4) find that people over 65 years of age represent 80% of COVID-19 hospitalizations. The older population is also at a much higher risk of mortality than the younger population (5, 6). Reports exposed a striking case fatality rate of 61.5% for critical cases in the older population (7).

In April 2020 United Nations published a report (8) recognizing older persons, people with extreme poverty, and minorities as high risk groups during the pandemic, which remained a major concern during the pre-vaccination period (9). Indeed, numerous metropolitan cities in the U.S. reveal that African American and Hispanic Americans comprise a disproportionate number of COVID-19 infections and mortality relative to their share of the population in the respective cities (10, 11). To this effect, Dobin et al. (12) showed that the COVID-19 infection rate is four-fold for the Non-Hispanic Black (NHB) and Hispanic population in New York state. Adhikari et al. (13) observe income inequality alone can not explain the racial and ethnic disparities in COVID-19 infections and deaths. Finally, Drefahl et al. (14) show that a low educated population is at higher risk of dying due to COVID-19. In light of these findings, we chose to include population density, age, minority population percentage, poverty, and education level as sociodemographic factors for counties considered in our work.

Understanding the impact of these selected demographic risk factors and air pollutants within a country is important. More rigorous investigation of these socioeconomic inequalities is needed to understand sociodemographic risk factors' association with COVID-19 severity and fatality. Thus, a multivariable study can help analyze the contrasting impact of demographic risk factors and air pollutants to enumerate their contributions.

### Effect of air pollutants on COVID-19 spread and prognosis

Air pollution exacerbates many of the known comorbidities responsible for hospitalization and fatality due to COVID-19. Kampa and Castanas (15) show that chronic exposure to air pollutants is associated with respiratory and heart conditions, such as chronic bronchitis, hypertension, ischemic heart disease. On the other hand, recent studies have revealed that fatality from COVID-19 is highly associated with chronic obstructive pulmonary disease (COPD), asthma, diabetes, hypertension, obesity (16–18).

Fine particulate matter with a diameter <2.5 μm, referred to as PM_2.5_, are air pollutants that can penetrate the lung, irritating the alveolar wall (19). Thus, PM_2.5_ pollution can lead to an impaired respiratory system. The adverse role of PM_2.5_ as the underlying contributor to respiratory diseases is noteworthy (20, 21). Several studies have determined that long-term exposure to PM_2.5_ adversely affects the respiratory and cardiovascular systems and increases mortality risk, as observed for COVID-19 (22, 23). Indeed, Wu et al. (24) show that an increase of only 1 μ*g*/*m*^3^ in PM_2.5_ is associated with an 8% increase in the COVID-19 fatality rate.

Nitrogen dioxide (NO_2_) is another toxic pollutant prevalent in urban areas that enters the atmosphere due to fossil fuel combustion from vehicles, power plants, and natural processes. High concentrations of NO_2_ in the environment damages the human respiratory system (25). Many studies have shown that elevated exposure to NO_2_ causes hypertension, COPD, cardiovascular diseases, lung injury, even diabetes (26). A high concentration of NO_2_ under ultraviolet light of around 400 nm generates ozone (O_3_) as a secondary pollutant [(27), p. 92]. Indeed, a review by Ali and Islam (28) demonstrated that both short-term and long-term exposure to air pollution especially PM_2.5_ and NO_2_ may contribute significantly to higher rates of COVID-19 infections and mortality. However, Ali and Islam (28) also call for further research with confounding factors such as age and population density.

Burning fossil fuels by power plants and other industrial facilities constitute the primary source of sulfur dioxide (SO_2_), one of the air pollutants of concern (29). Wong et al. (30) show that SO_2_ pollution increased the risk of hospitalization due to respiratory disease. Increased level of exposure to O_3_ is associated with decreased function of airways (31). Turner et al. (32) find that long-term O_3_ exposure contributes to the risk of respiratory mortality.

Thus, the study of air pollutants which aggravates the infection and fatality rates for COVID-19 disease has increasingly become relevant, for which the supporting evidence is mounting. The understanding of their impact will help to make informed decisions at all levels. Considering these recent findings, we aim to quantify the role of PM_2.5_, NO_2_, SO_2_, and O_3_ in COVID-19 infections and fatality.

### Geographical factors influencing in the pandemic

Early reports have shown that the geographical patterns of COVID-19 spread and fatality within and among different regions of a country closely align with local levels of air pollutants (33). In addition to temporal studies (34), spatiotemporal assessment of air quality can help identify reasons for local transmission of this pathogen, specific populations who could be at higher risk, and critical factors that facilitate the spread. To date, spatiotemporal studies are limited. In case of U.S., it is observed that northeastern part of the country experienced more cases and deaths compared to other regions during the initial phase of the pandemic (35). At present, only area-level counts for COVID-19 infection and fatality data are publicly available.

### Objectives

We aim to advance the understanding of the association of COVID-19 infection and fatality rates with demographic risk factors and selected air pollutants for the entire population of the U.S. through a comprehensive framework. In this work, we investigate the following questions:

i. To what extent do the demographic variables, such as age, socio-economic status, and ethnicity, impact COVID-19 transmission and fatality?
ii. Is there a significant difference in the concentration of air pollutants, such as PM_2.5_, NO_2_, SO_2_, and O_3_, in the counties with high COVID-19 infection rates compared to counties with low infection rates?
iii. On the temporal effect, does chronic exposure of the pollutants remain constant or differ as the pandemic enters the later Phase.

We note that the factors we examine are by no means comprehensive, and several of them are interrelated. Identifying region-wide variations influenced by significant risk factors and underscoring their interactions will help to make strategies to protect those in the most vulnerable counties requiring urgent care.

## Materials and methods

### Data collection

#### Data sources

We used publicly available data from New York Times (36) for COVID-19 infections and fatality. The air pollution data was obtained from United States Environmental Protection Agency (37). The sociodemographic information was acquired from the Hopkins Population Center (38). The data sources are listed in Table 1.

**Table 1:** Publicly available data sources used in this study.

**Data**	**Source**
Covid-19 cases and deaths	Coronavirus (COVID-19) data
	(https://developer.nytimes.com/covid)
Population estimates and demographics 2018	Hopkins population center (HPC)
	(https://popcenter.jhu.edu/data-hub)
Air pollutants data	Air quality system (AQS) API
	(https://aqs.epa.gov/aqsweb/documents/data_api.html)

#### Defining Phase **1** and Phase **2** of the pre-vaccination efforts

In this work, we wanted to understand the spread of the COVID-19 pandemic before the vaccine distributions. The Federal Drug Administration approved the first mRNA vaccine for emergency use in December 2020 (39). However, the vaccine distribution began in January 2021 (40). Thus, we considered the time period of January 1, 2020 to December 31, 2020 for our analysis. The pre-vaccination period of the pandemic could be divided into two phases. The initial phase was marked by a sharp rise in COVID-19 deaths, reaching the peak in its 7-day average in mid-April and a trough at the end of June 2020 (41). Moreover, in the U.S. the number of new cases decreased to around 20,000 the month of June 2020, before increasing again (36). Thus, we considered the period of January 1, 2020 to June 30, 2020 as the Phase **1** of the pandemic. We define the period July 1, 2020 to December 31, 2020 as the second phase.

#### Response variables

We wanted to study the spread of COVID-19 infection, and fatality due to COVID-19. To be able to make a comparison between counties with varying populations, we used infection rate for a population of 10,000 people instead of the actual number of infections. We defined this variable as follows:


$$\begin{array}{l} \text{Infection rate}\\ \;=\frac{\text{Number of COVID-19 infections in the county}}{\text{Population of the county}}\times 10,000. \end{array}$$

Similarly, we defined the fatality rate per a population of 10,000 for a county as follows.


$$\begin{array}{l} \text{Fatality rate}\\ =\frac{\text{Number of deaths due to COVID-19 in the county}}{\text{Number of COVID-19 infections in the county}}\times 10,000. \end{array}$$

We used the fatality rate as opposed to the mortality rate (deaths per population) in our work, since the fatality rate captures the effectiveness of the response to COVID-19 infected population. We obtained the COVID-19 infection and mortality data from New York Times (36), and the county population data from Hopkins Population Center (38). Note that the infection and fatality rates for a given county differ in Phase **1**, Phase **2**, and when all year data was considered.

#### Explanatory variables

We obtained countywise demographic data for population density, ethnicity, age, and education from the United States Census Bereau (42). We used six demographic variables in this work defined as follows:

i. Population density : population of a county per area of the county in square miles.
ii. Age 65+ : percentage of people who are of age 65 and more in the county.
iii. African Americans : percentage of African Americans in the county.
iv. Hispanic Americans : percentage of Hispanic Americans in the county.
v. Poverty: percentage of people living under the poverty line in the county.
vi. High school or less: percentage of people with maximum education of high school in the county.

Furthermore, we studied impact of the following pollutants as the four of the explanatory variables in this work:

vii. PM_2.5_ (μ*g*/*m*^3^),
viii. NO_2_ (ppb),
ix. SO_2_ (ppb),
x. O_3_ (ppb).

We acquired the weekly and annual levels these pollutants from the EPA's Air Quality System (AQS) database (37) through 2015 to 2020.

#### Zone **A** vs. Zone **B** counties and impact of non-availability of the pollutant data

Since the pandemic affected larger counties in the initial stages of the pandemic (43), we considered only counties with population of 100,000 or more. There were 593 such counties in the U.S. which we sorted based on the infection rate. We categorized the top 200 counties with most infection rates as Zone **A** counties, the rest were labeled as Zone **B**. Since the infection rate may differ from Phase **1** to Phase **2**, counties which were in Zone **A** during the Phase **1** may not remain in Zone **A** in the second Phase. The limited number of EPA sampling sites prevented the acquisition of pollutants data from all 593 counties. Thus, for Phase **1**, pollutant data was available for 64 counties out of 200 counties from Zone **A** and 54 counties out of 393 counties from Zone **B**. Similarly, for Phase **2**, we obtained the pollutant data for 42 counties from Zone **A** and 76 counties from Zone **B**. For all year, we obtained the pollutant data for 45 counties from Zone **A** and 73 counties from Zone **B**.

## Statistical analyses

### Description of the study variables

The data for this study are county level demographics and air pollutants. This data is available at a github repository. We computed the medians, first, and third quartiles of the zone-wise characteristics of six demographic variables and four air-pollutants selected for this study for Phase **1** and Phase **2**, and the whole year. After confirming the normality assumptions, we performed *T*-tests to check for any statistically significant differences in the demographic risk factors and environmental variables between Zones **A** and Zone **B** during both phases and throughout the entire year.

### Correlation analysis

For Zones **A** and **B** counties for both Phase **1** and Phase **2**, we summarized Pearson's correlation coefficients between infection and fatality rates and the corresponding *p*-values in correlation matrices. All analyses used two-sided statistical tests, and *p* < 0.1 was considered significant.

### Autoregressive integrated moving average (ARIMA) model

We used auto-regressive integrated moving average [ARIMA (*p, q, d*)] models (44, 45) to analyze how concentrations of PM_2.5_, SO_2_, NO_2_, and O_3_ differed between Zones **A** and **B** over the 6 year time period from 2015 to 2020. To this effect, we used the time-series of monthly data collected by the EPA from 2015 to 2020 to obtain predicted estimates of these pollutants. We used the Augmented Dickey-Fuller (ADF) unit-root test (46) to verify that the time-series was not stationary. We built the models with varying orders of *p, q*, and *d* for the pollutants PM_2.5_, SO_2_, NO_2_, and O_3_. We used Akaike information criterion (AIC) to evaluate the goodness of fit for the models. We plotted the fitted values of each model and the corresponding 95% confidence bands for each zone. We then compared these fitted values of the concentrations the pollutants in Zone **A** vs. those in Zone **B** for Phase **1**, Phase **2**, and for all year data. We employed autoregressive neural networks and exponential smoothing techniques and compared the predicted accuracy of ARIMA models by computing the commonly used statistic “root mean squared errors (RMSE)” (45, 47).

### Tests for significance

Since the normality assumptions were satisfied, we used two-sided *T*-tests for statistical comparisons between demographic parameters and pollutants for both Zones during Phase **1**, Phase **2**, and all year.

### Regression analysis

**Figure 1:**
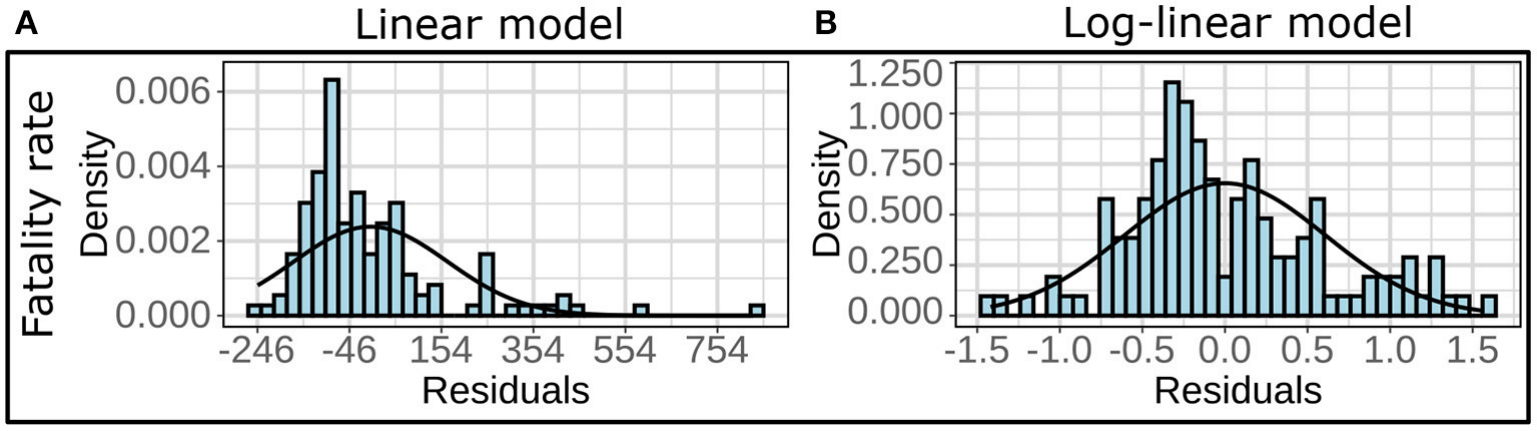
**(A)** Histograms and the fitted normal curves of the residuals for multivariate linear regression and **(B)** multivariate log-linear regression. The linear models are based on fatality rate as the response variable for the all year (2020) data in Zone B.

We employed multivariate linear regression models to demonstrate the role of the explanatory variables on specific aspects of COVID-19 burden, namely infection rate and fatality rate. To this effect, we considered each county belonging to Zone **A** or Zone **B** as a data point and observed that the residuals for these linear models did not follow the normality assumptions (see Figure 1A). However, the residual for the regression models on log-transformed responses variables satisfied the normality assumptions (see Figure 1B). The residuals for the log-linear models are provided in Supplementary Figures 1, 2. Thus, we implemented a logarithmic (log) transformation on the response variables to conform to the normality of the distributions of the residuals and built 12 linear models using infection rates and fatality rates as response variables. Four pollutants and six demographic risk factors were used as predictors for all twelve models (see Section Explanatory variables). By measuring the variance inflation factor (VIF), which assesses the inflation in the variances of independent parameters due to interdependence to avoid unstable and incorrect estimation of regression coefficients (48), multicollinearity between the explanatory variables was evaluated.

We set an upper cut-off value for VIF at 5 for the explanatory variables. We used the following procedure outlined in Athavale et al. (49) with a cut-off of VIF = 5 to construct our final models for infection and fatality rates for each of two zones and two phases.

Step 1: Compute the VIF for each explanatory variable in the model. If all the VIFs are <5, we declare this to be the final linear model.
Step 2: If an explanatory variable has a VIF of more than 5, we remove the explanatory variable with the largest VIF. If there are more than one explanatory variables with VIF within 5% of the maximum VIF, we remove the variable that leads to a model with the highest *R*^2^.
Step 3: We construct the linear model with the remaining explanatory variables.
Step 4: Go to Step 1.

After building the linear models, we verified the residuals' normality and homoscedasticity assumptions.

### Coding language and libraries used

Analyses were performed using version 3.7.12 of the Python programming language and 4.0.0 of the R programming language. The Python packages used here are statsmodels 0.10.2, matplotlib 3.2.2, scipy 1.4.1, numpy 1.21.5, and pandas 1.3.5. The R libraries used here are, readxl, dplyr, tidyr, ggplot2, ggpubr, MASS, and car.

## Results

### Descriptive statistics

**Figure 2:**
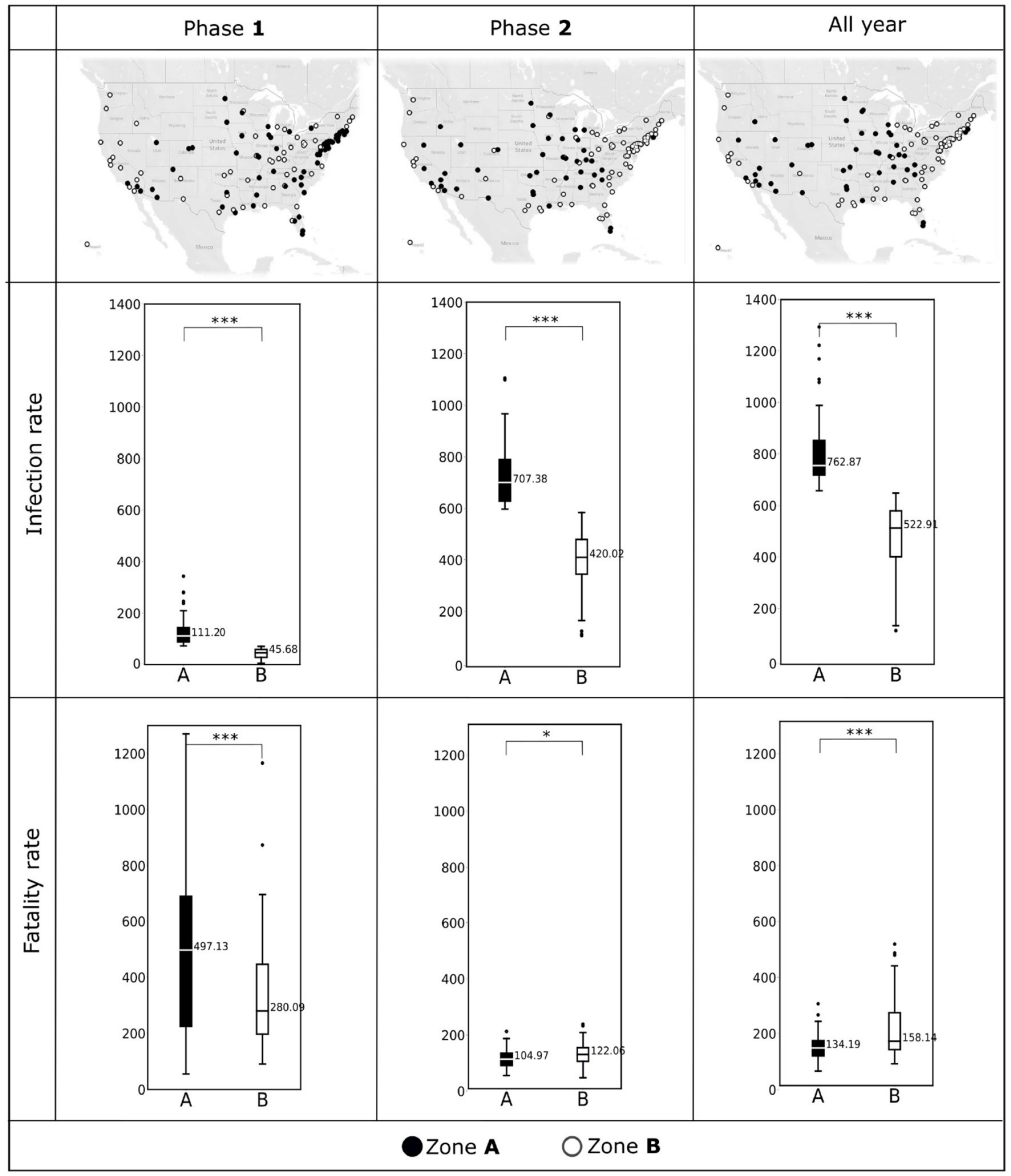
**(Top)** The counties included in Zone **A** and Zone **B** during Phase **1** (January, 2020–June, 2020), Phase **2** (July, 2020–December, 2020), and all year (2020) are shown in the U.S. maps. **(Middle)** Box plots showing COVID-19 infection rates (per 10,000 population) observed in Zone **A** and Zone **B** during Phase **1**, Phase **2**, and all year. **(Bottom)** COVID-19 fatality rates (per 10,000 infections) observed in Zone **A** and Zone **B** during Phase **1**, Phase **2**, and all year. Horizontal black lines represent median values. *p < 0.1; ***p < 0.01; NS, not significant.

As discussed in Section Zone **A** vs. Zone **B** counties and impact of non-availability of the pollutant data, we separated the counties in two zones, Zone **A** and Zone **B**. We display the map of these counties for Phase **1**, **2**, and all year in the first row of the Figure 2.

**Figure 3:**
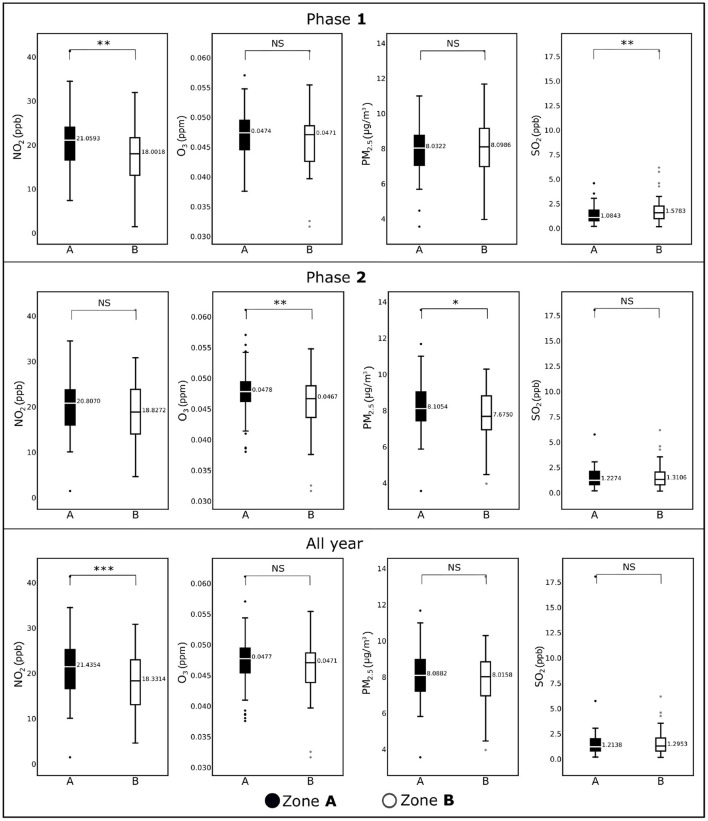
Box plots showing average concentrations of NO_2_, O_3_, PM_2.5_, and NO_2_ for the counties included in Zone **A** and Zone **B** for Phase **1** (January, 2020–June, 2020), Phase **2** (July, 2020–December, 2020), and all year (2020). Horizontal black lines represent median values. *p < 0.1; **p < 0.05; ***p < 0.01; NS, not significant.

**Figure 4:**
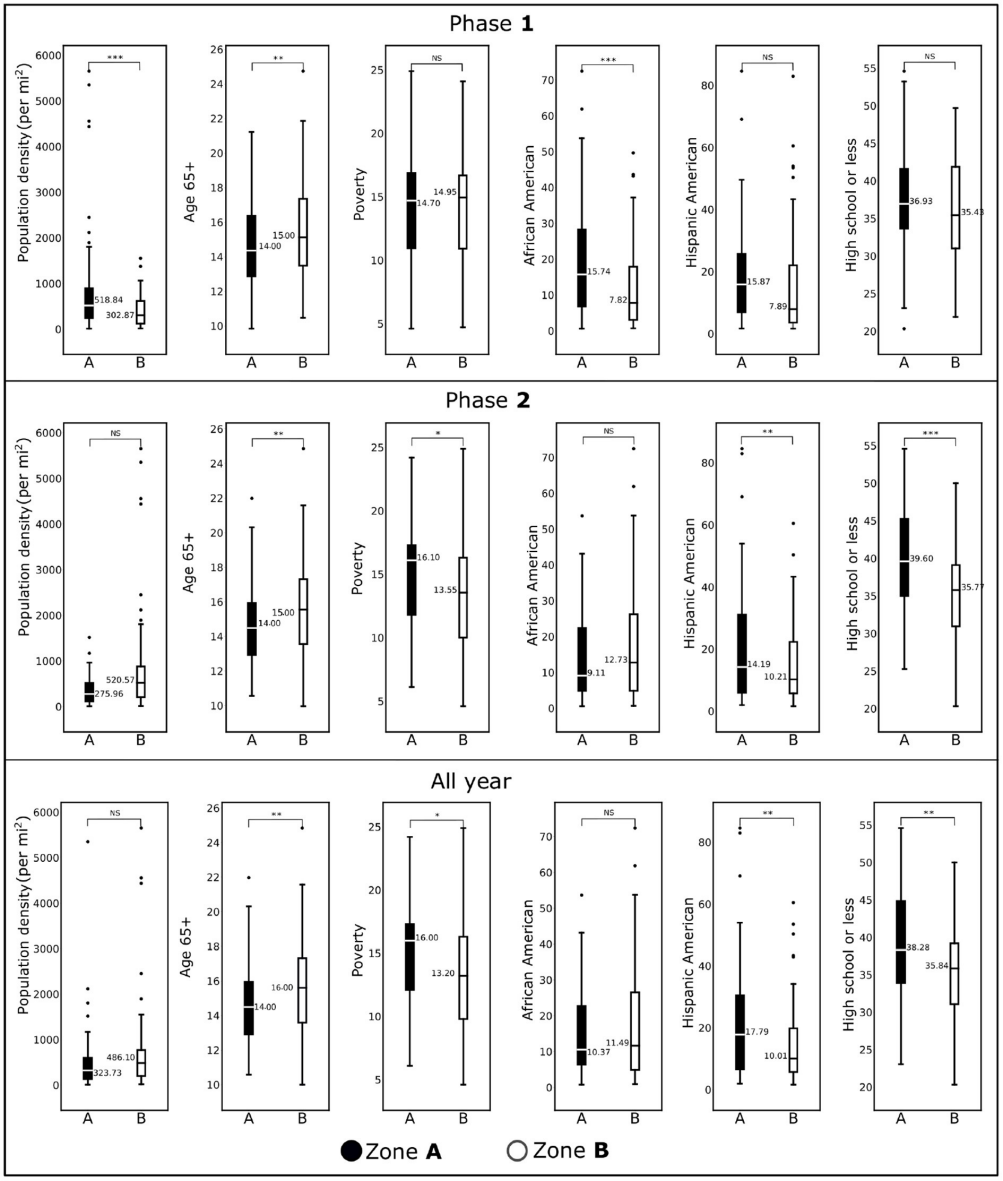
Box plots showing demographic variables for the counties included in Zone **A** and Zone **B** for Phase **1** (January, 2020–June, 2020), Phase **2** (July, 2020–December, 2020), and all year (2020) are shown. Horizontal black lines represent median values. *p < 0.1; **p < 0.05; ***p < 0.01; NS, not significant.

We depict the boxplots for infection rates for the two phases and for the entire year are in the second row of the Figure 2. Similarly, the third row of Figure 2 shows the boxplots for fatality rates for the two phases and for the entire year. We summarize the descriptive statistics for the explanatory variables in Figures 3, 4.

### Results of the correlation analyses

Supplementary Tables 1–6 show the pairwise Pearson's correlation coefficients to quantify the association between the response and explanatory variables for Zones **A** and **B** counties for Phases **1**, **2**, and for all year.

The tables shows two-sided *p*-values for each of the correlation tests, and *p* < 0.1 was considered significant. During the first Phase, we note that the pollution level was not positively correlated to the fatality rate. However, during the second Phase all the pollutants are positively correlated (with *p* < 0.1 or less) to the infection rates in the Zone **B** counties. This pattern can not be seen if we look at the all year data for Zone **A** (see Supplementary Table 5). We observe that the percentage of population of age 65 or more is consistently strongly correlated with fatality rate. We also see that the Hispanic population is significantly and positively correlated to the infection rate in both Zones, during both Phases, as well as in the all year data. Notably the education level of the counties is inversely related to the fatality during the Phase **2**, but not during the first Phase.

### Results of the ARIMA

**Figure 5:**
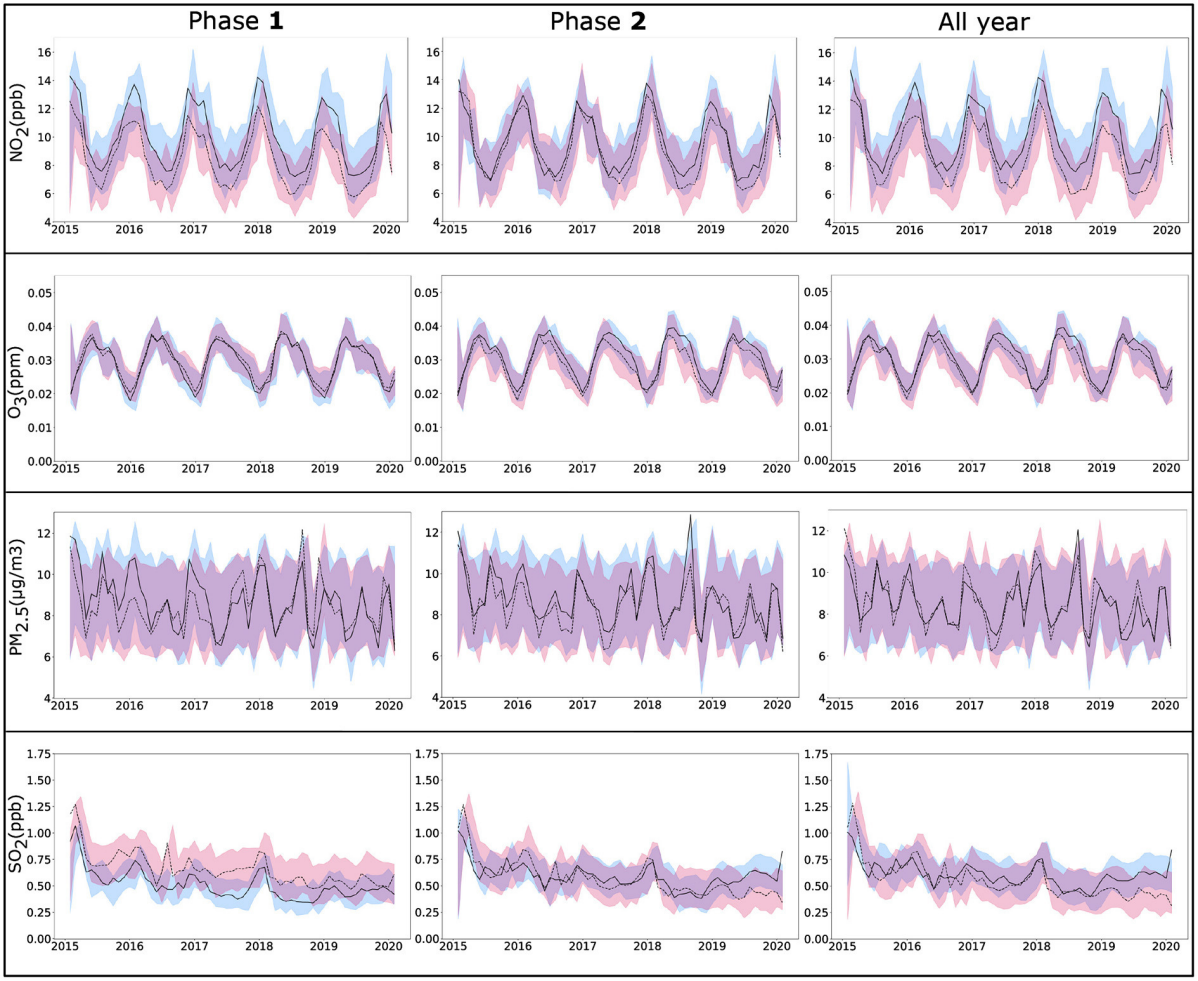
ARIMA time series analysis of four pollutants NO_2_, O_3_, PM_2.5_, and SO_2_, from monthly EPA data (2015–2020) for the counties selected in Zone **A** (solid line) and Zone **B** (dotted line) for Phase **1** (January, 2020–June, 2020), Phase **2** (July, 2020–December, 2020), and all year (2020). Lines show fitted values for pollutants with 95% confidence bands. ARIMA, autoregressive integrated moving average; EPA, environmental protection agency.

The Augmented Dickey-Fuller (ADF) unit-root test (46) confirmed the stationarity of the time series. Figure 5 shows ARIMA models and the 95% confidence bands for the pollutants PM_2.5_, NO_2_, SO_2_, O_3_ for the two zones and for Phase **1**, Phase **2**, and for all year data. The AIC values of the models for all conditions were low and comparable (−555.15, 202.53). The pattern was similar for Zone **A** and Zone **B** for the pollutants for pollutants PM_2.5_, NO_2_, and O_3_ where considerable overlap of the confidence bands were observed with periodic temporal variation between the models. In contrast, values depicted larger separation between Zone **A** and Zone **B** during the Phase **1** for the models which predicted SO_2_. Additionally, exponential smoothing and autoregregressive neural network yielded RMSE values comparable to ARIMA model (see Supplementary Table 13) for the pollutants PM_2.5_, NO_2_, O_3_, SO_2_, for the two zones and for Phase **1**, Phase **2**, and for all year data.

### Results of the tests of significance

The first row of Figure 2 shows the distribution of the counties in Zone **A** and Zone **B** for the two phases and the whole year of 2020. We see a movement of the counties from Zone **A** and Zone **B** as time progresses. Notably, most counties on the east coast which categorized as Zone **A**, switched to Zone **B** in the second Phase. The boxplots depicted in the second and the third rows of Figure 2 demonstrate the statistically significant difference (*p* < 0.01) between the two zones with respect to the infection rates and fatality rates. Figure 2 indicates that the differences between Zone **A** and Zone **B** are statistically significant for the infection rates during Phase **1**, Phase **2**, and over the entire year (*p* < 0.01). The same is true for fatality rates; however, for Phase **2** the fatality rates were comparable between the two zones (*p* < 0.1).

We display the difference between the two zones with respect to the pollutants and sociodemographic variables in Figures 3, 4 respectively. We observe in Figure 3 that the NO_2_ levels are significantly different (*p* <.05) in Zone **A** and Zone **B** counties during Phase **1**. This difference diminished during Phase **2**. However, for other pollutants the distributions of the data for Zone **A** and Zone **B** remained more or less the same with the exceptions of except for O_3_ during Phase **2** (*p* < 0.05), PM_2.5_ during Phase **2** (*p* < 0.10), and SO_2_ during Phase **1** (*p* < 0.01). Interestingly, NO_2_ remained the only pollutant that showed significant statistical differences for the entire year's data (*p* < 0.01), where Zone **A** was significantly higher than Zone **B**.

For population density, the difference between Zone **A** and Zone **B** for Phase **1** was statistically significant (*p* < 0.01), whereas for Phase **2**, there was no statistical difference. We observe the same trend for the African American population in two phases. We observed no statistical differences for these two demographic factors for the whole-year data. The percentage of the population with age over 65 years remained significantly different for both zones over two phases and the entire year. There was no statistical difference between Zone **A** and Zone **B** for the percentage of the Hispanic American population during Phase **1**. In contrast, we discovered statistically significant differences during the second phase and when the entire year data were analyzed. For the risk factor “Poverty” (the percentage of population below the poverty line), we observed either no statistical differences (Phase **1**) or marginally different (Phase **2** and entire year data). We discovered an interesting trend for the variable “percentage of the population with high school or less”: during Phase **1** there was no statistical differences between the two zones (*p*>0.1), whereas they were significantly different (*p* < 0.01) during Phase **2** and for the entire year's data.

### Results of linear models with infection and fatality rates as response variables

In Table 2, we present the *p*-values of the explanatory variables in linear models with log-transformed infection rate and fatality rate as response variables for each case. The *p*-values <0.05 corresponding to the positive coefficients are boldfaced for easy interpretation of the model. The complete details of all the 12 models are provided in Supplementary Tables 7–12.

**Table 2:** The *p*-values of the explanatory variables generated from the linear regressions models outcomes with (log-transformed) infection and fatality rates as response variables for Phase **1**, Phase **2**, and all year data, developed for Zone **A** and Zone **B**, respectively.

	**Phase 1**				**Phase 2**				**All year**			
	**Zone A**		**Zone B**		**Zone A**		**Zone B**		**Zone A**		**Zone B**	
	**Infections**	**Fatality**	**Infections**	**Fatality**	**Infections**	**Fatality**	**Infections**	**Fatality**	**Infections**	**Fatality**	**Infections**	**Fatality**
**Sociodemographic**												
**variables:**				
Population density	**<0.001**	**0.002**	0.873	0.310	0.526	0.053	0.077	0.471	**0.023**	0.097	0.673	**0.035**
Age 65+	0.312	**<0.001**	0.883	**0.025**	0.217	**0.004**	0.544	**0.031**	0.017	**0.015**	0.149	0.083
African American	0.727	0.563	**0.010**	0.765	0.209	0.134	0.217	0.451	0.126	0.581	**0.013**	0.777
Hispanic American	0.911	0.014	0.485	0.300	0.283	0.072	0.074	0.483	0.372	0.900	0.244	0.270
Poverty	0.210	**0.045**	0.239	0.617	0.553	0.864	0.147	0.129	0.154	0.684	0.470	0.230
High school or less	**0.005**	**0.008**	0.703	0.289	0.314	0.181	0.238	0.075	0.741	**0.046**	**0.005**	**0.005**
**Pollutants:**												
PM_2.5_	0.172	0.787	0.711	0.698	0.771	0.066	0.189	0.542	0.364	0.109	0.643	0.116
NO_2_	0.580	**0.015**	**0.005**	**0.046**	0.934	0.615	0.578	0.828	0.440	0.090	0.953	**0.009**
SO_2_	0.605	0.456	**0.006**	0.408	0.448	0.794	0.174	0.674	0.749	0.763	0.274	0.267
O_3_	0.912	0.379	0.930	0.750	0.050	0.901	**0.003**	0.494	0.067	0.361	**<0.001**	0.719
Sample size (*n*)	64	64	54	54	42	42	76	76	45	45	73	73
Coefficient of												
determination (*R*^2^)	0.37	0.57	0.53	0.32	0.31	0.58	0.47	0.29	0.44	0.48	0.60	0.43

The *p*-values <0.05 corresponding to the “positive” coefficients are boldfaced for easy interpretation. For detailed models see Supplementary Tables 7–12.

#### All year models

For the models using the entire year's infection rate, population density (*p* < 0.05) was the only positively significant in Zone **A**. African American population (*p* < 0.05), O_3_ (*p* < 0.01), and “high school or less” (*p* < 0.01) were significant for Zone **B** infection rates. Old age and less education were both significant for Zone **A** fatality rates with *p* < 0.05. On the other hand population density, less education, and NO_2_ pollution were statistically significant for Zone **B** fatality rates. However, when we view at the data during Phase **1** and Phase **2** separately we get a granular insight into the pandemic's progression.

#### Phase 1 models

During the Phase **1**, with infection rate as outcome variable, demographic factors, “population density” (*p* < 0.01) and “high school or less” were the strongest and statistically significant parameters (*p* < 0.01) for Zone **A** which generated the coefficient of determination (*R*^2^) of 0.37. On the other hand, in Zone **A** counties, population density, old age, and population with high school or less were the most significant (*p* < 0.01) risk factors for fatality rates. The NO_2_ pollution, and poverty were also strong predictors (*p* < 0.05) of fatality with *R*^2^ = 0.57. The percentage of African Americans and NO_2_ remained statistically significant (*p* < 0.01) for Zone **B** infection rates. Whereas, old age and NO_2_ were statistically significant (*p* < 0.01) for Zone **B** fatality rates.

#### Phase 2 models

Old age was a significant factor (*p* < 0.01) for the fatality rate in both the Zone **A** and Zone **B** counties during the second Phase. For the Zone **B** infection rates O_3_ was the most significant contributor with *p* < 0.001.

## Discussion

One of the surprising revelations of our work is that the COVID-19 data reveals several hidden features when it is segmented spatiotemporally. In this work, we categorized highly populated counties in the U.S. into two Zones **A**, and **B**, based on their infection rates. Zone **A** counties had a higher infection rate than Zone **B** counties. A trough separates phases **1** and **2** in the COVID-19 deaths.

### Spatiotemporal wave of the pandemic

We observe the pandemic spreading spatially in the first row of the Figure 2. In Phase **1**, the highest infected counties were in the northeast coast of the U.S. and the southern California regions. This phenomenon is because most initial COVID-19 cases were travel-related. These counties became hot-spots due to the high population densities. We also see from the third row of the Figure 2 that the fatality in Phase **1** remained high despite the low infection rate. However, as time passed, we noted that the spatial part of the wave of the infections traveled inward, and most northeastern counties were no longer categorized as highly infected in Phase **2**. We can not observe this inward traveling spatial wave from the cumulative data from 2020. Since we categorized the zones based on infection rates, as expected, we see a significant difference between the infection rates of the two zones. However, We see that the significant difference (*p* < 0.01) between the two zones' fatality rates in Phase **1** reduces in the second Phase (*p* < 0.1). Again this reduction in the fatality rate can not be observed in all year data. The “exact opposite” conclusion could be drawn from the all-year data, where we notice that the fatality rates in Zone **B** are significantly more than that in Zone **A**.

### Spatiotemporal pandemic wave and pollution levels

Among the air pollutants considered in this study, differences observed between the counties of two zones were most prominent for NO_2_ (Figure 3), which also demonstrated a significant association with COVID-19 fatality. The inter-zonal difference of NO_2_ level was higher during the Phase **1** of the pandemic compared with Phase **2**, and it remained significant when both phases considered together. In contrast, all-year data for other pollutants did not demonstrate any significant difference between the counties from two zones, although significant difference was noted for SO_2_ in the Phase **1**, and for PM_2.5_ and O_3_ in the Phase **2**. The ARIMA plots in Figure 5 corroborate these findings, where we observe a historical difference in NO_2_ and SO_2_ levels between Zone **A** and Zone **B** counties during Phase **1** with substantial reduction of the difference during Phase **2**. The predictive accuracy of the ARIMA model was further confirmed by exponential smoothing and autoregressive neural network models. Interestingly, the regression analyses (Table 2) revealed that NO_2_ level is a significant factor driving the fatality rates only during Phase **1**, but not in Phase **2**. A number of studies investigating the relationship between NO_2_ in air and COVID-19 reported a variable degree of association with infection and fatality (28, 50). Exposure to NO_2_ is known to cause lung injury and is associated with elevated risk of developing asthma and exacerbation of chronic lung diseases such as asthma, bronchitis, and COPD (50). As a consequence, the lungs can be more susceptible to infections along with more adverse outcomes, including fatality. Additionally, *in vitro* and animal model studies have shown NO_2_ to upregulate the expression of Angiotensin Converting Enzyme 2 (ACE2), which provides binding to the virus spike protein (51, 52). The upregulation of ACE2 expression in lung epithelial cells following NO_2_ exposure is proposed to further contribute to its adverse impact on COVID-19 by facilitating the virus attachment (53). The reason for a stronger association of NO_2_ with COVID-19 during the Phase **1** is not well-understood, but could partly be attributed to a larger pool of susceptible population along with higher disease fatality rate during the earlier stage of the pandemic. The other air pollutants considered in this work (PM_2.5_, SO_2_, and O_3_) demonstrated a weaker association with COVID-19 during both Phase **1** and Phase **2**, or the whole year. Prior studies investigating the association of these pollutants with COVID-19 infection or fatality show a high degree of variation in their reported results, suggesting a strong dependency on the selection of study population or method of analysis (28, 50, 54). In this work, we used pollutant data obtained from EPA monitoring sites. While EPA measurements provide more accurate estimates of pollutant levels compared with other available techniques such as estimates from satellite images or low-cost sensors, the data can be obtained only from a limited number of measurement sites across the U.S. Therefore, in our analysis, we could only include the counties with available EPA measurement sites, leading to the exclusion of a considerable fraction of counties. Such exclusion can potentially contribute to some of the differences we observed with previous studies; future work involving accurate pollutant measurements along with a greater coverage of counties would provide a deeper understanding of the effects of the pollutants on COVID-19.

### Spatiotemporal pandemic wave and sociodemographic variables

Páez-Osuna et al. (3) found that high population density led to high COVID-19 mortality. We corroborated this finding with our linear model. However, our regression analysis (Table 2) reveals that this is more significant (*p* < 0.01) in the first Phase of the pandemic than the later Phase. In fact, from the last row of Figure 4 we observe no significant difference between the population density of the Zone **A** and Zone **B** counties from the all-year data. However, from the first row of Figure 4, we note that there was a significant difference between the two Zones during Phase **1**. This discrepancy is again due to the Spatial nature of the pandemic wave. We observe an interesting phenomenon regarding the percentage of people with High School or less education. We found no statistically significant difference in the percentage of people with high school or less education in Zone **A** and Zone **B**, during Phase **1**, but there was a significant difference in Phase **2**. However, our regression model (Table 2) shows that less education was one of the main risk factors in Zone **A** counties during Phase **1**. This apparent contradiction indicates that the even though the percentage of less educated people was similar in Zone **A** and Zone **B** during Phase **1**, having less education was a risk factor in Zone **A** counties when the pandemic just began. This could be because the population with less education were compelled to do jobs with more exposure to people. In contrast, people with more education could work remotely, thus avoiding exposure to infections. From a public policy point of view, this underscores the importance of access to high-quality affordable education. Mueller et al. (4) found that people over 65 were at a high risk in the COVID-19 pandemic. We confirmed this finding with the regression models (Table 2) where we note that older age was a significant risk factors for infections and fatality.

When we studied the linear model using all-year data, we find that the Hispanic population percentage is not a risk factor for infections and fatality. However, the percentage of the Hispanic population is a significant risk factor for the fatality rate in Zone **A** during Phase **2** and the infection rate in Zone **B** during Phase **2**. This finding also supports findings of Athavale et al. (49) where Hispanic population was found to be at a greater risk in the COVID-19 pandemic. We would like to draw the attention to an interesting finding in model for the fatality rate in Phase **1** for the Zone **A** counties. From the results of the regression model in Supplementary Table 7, we see that the coefficient for the Hispanic American is negative. This finding is consistent with Bassett et al. (55), and could be the result of a younger demographics of the Hispanic population. The linear model for Zone **A** during Phase **1** suggests that poverty was a significant risk factor driving the fatality rate higher, a conclusion that would be missed if we had considered only all-year data. Finally, the importance of education is highlighted again because less education was a significant risk factor during Zone **A**, which could be because the people with less education did not have the privilege of working from home during the pandemic.

### Limitations

In our analysis, We used high-quality environmental data obtained from the EPA. However, one of the main challenges that we faced in this work was the lack of availability of environmental data from most counties in the U.S. Due to this issue, we had to limit our work to 128 counties from the 593 initially considered. This limitation also hampered our attempts to study the interactions between the air pollutants and the sociodemographic variables, which we plan to pursue in future research. The other issue we faced was the problem of separating the two Phases of the pandemic. As our research shows, the pandemic moved inward from the coastal areas as time passed. Hence, separating the Phases into two parts is only an approximation. However, the analysis is still helpful because it demonstrates the need to separate the data spatially and temporally.

### Practical implications of the study

Our work points to several policy steps that we can take to mitigate the effects of the pandemic. Since the older population is at significantly higher risk, any new medical interventions need to begin with the older population. Indeed, the vaccines were administered to the older population in the U.S. We also found that the pandemic's impact was more severe on minority population. Thus, as pointed out by UNSDG (8) efforts should be made to make healthcare accessible to underprivileged and minority population. To this effect, in the U.S., vaccines were provided at no cost; as well as the first preference was given to people at high-risk of infection, such as older population, immunocompromised people, and essential workers. However, we discovered that the counties with higher population density were highly vulnerable during the first Phase of the pandemic. Hence, we would recommend that the medical interventions start with places with high population density to mitigate the spread of infection. We also observed that the minority population was highly vulnerable in this pandemic.

One of the main findings of our work is that during the initial Phase of the pandemic, people with less education were most at risk of both infection and fatality. Rather unsurprisingly, we see a strong correlation (Supplementary Tables 1–6) between poverty and less education. Providing access to quality education should be an urgent priority in the U.S. Indeed, counties with most people with tertiary education (56), i.e., Ireland, Canada, and South Korea saw fewer mortality (1,359, 990, 365 deaths per million, respectively) compared to the U.S. (3,023 deaths per million https://www.worldometers.info) as of this writing. Thus, education level played a crucial part in lower fatality. Having access to high quality education, irrespective of the socioeconomic status is not only a way to upward mobility but is essential in saving lives.

## Conclusion

The COVID-19 pandemic exhibited temporal, as well as spatial variation in the U.S. One of the main realizations of this paper is that studying the pandemic over a larger time period, such as 1 year, can result in missing some important features of the pandemic data. However, these features are revealed when we segment the data spatiotemporally into small parts. In our work, we wanted to study the infection and fatality rates in large counties before the vaccination efforts started. To this effect, we divided this time period into two time periods, Phase **1** and Phase **2**. The counties were then divided into two zones based on the infection rates. We can see that the spatial part of the pandemic moved starting from the coastal ports of travel to the interior regions of the U.S. We also studied the difference between sociodemographic variables and air pollutants in these segments. When looked at data in strategically segmented way we found that the population density was a significant risk factor in the first Phase than the second Phase. Old age was found to be a risk factor in both Phases and both Zones. When we looked at the all year data, Hispanic population percentage was not found significant. However, looking at the spatiotemporally segmented data, a nuanced pattern emerged. The percentage of the Hispanic population was found to be significant risk factor in the Phase **2**. Having a population with high school or less education also emerged as a significant risk especially in the first wave of the pandemic. This could be because this group could not work from home in the initial phase of the pandemic due to the lack of social safety net. On the other hand, we found that poverty was a risk factor in the fatality rates in the Zone **A** counties in the Phase **1** only. The reason could be lack of access to scarce resources in regions with high levels of infections. However, since we also found a high correlation between lower education and poverty, we see that the investing in quality education is of utmost importance. Regarding the air-pollutants we found that the effects of the NO_2_ pollution were more significant in the first Phase of the pandemic than the later Phase. Moreover, Ozone was a significant factor predicting high infection rate in the Zone **B** counties. We suspect there is some interaction between the risk factors, but currently, the lack of complete EPA data prohibited further exploration in this direction. Finally, the emergence of structures in the spatiotemporal components of the data which is unseen in the aggregate data suggests further investigation of possibly finer spatiotemporal decomposition of the data.

## Data availability statement

Publicly available datasets were analyzed in this study. This data can be found at: https://github.com/chaipi-chaya/Spatiotemporal-analysis-on-COVID-19.

## Author contributions

CC and VK: data curation, formal analysis, visualization, and coding. PA: writing—review, conceptualization, methodology, editing, investigation, and analysis. TS: formal analysis. MB: validation and editing. SM: writing—original draft, conceptualization, methodology, formal analysis, and project administration. SS: supervision, conceptualization, validation, and editing. All authors contributed to the article and approved the submitted version.

## Conflict of interest

The authors declare that the research was conducted in the absence of any commercial or financial relationships that could be construed as a potential conflict of interest.

## Publisher's note

All claims expressed in this article are solely those of the authors and do not necessarily represent those of their affiliated organizations, or those of the publisher, the editors and the reviewers. Any product that may be evaluated in this article, or claim that may be made by its manufacturer, is not guaranteed or endorsed by the publisher.
