# Supplementary material for: COVID-19 in the U.S. during pre-vaccination period: Shifting impact of sociodemographic factors and air pollution
Source: Front Epidemiol. 2022 Oct 26;2:927189. doi: 10.3389/fepid.2022.927189 (PMC10910972; doi:10.3389/fepid.2022.927189)
Supplement: Supplementary file 1 [file Data_Sheet_1.PDF]

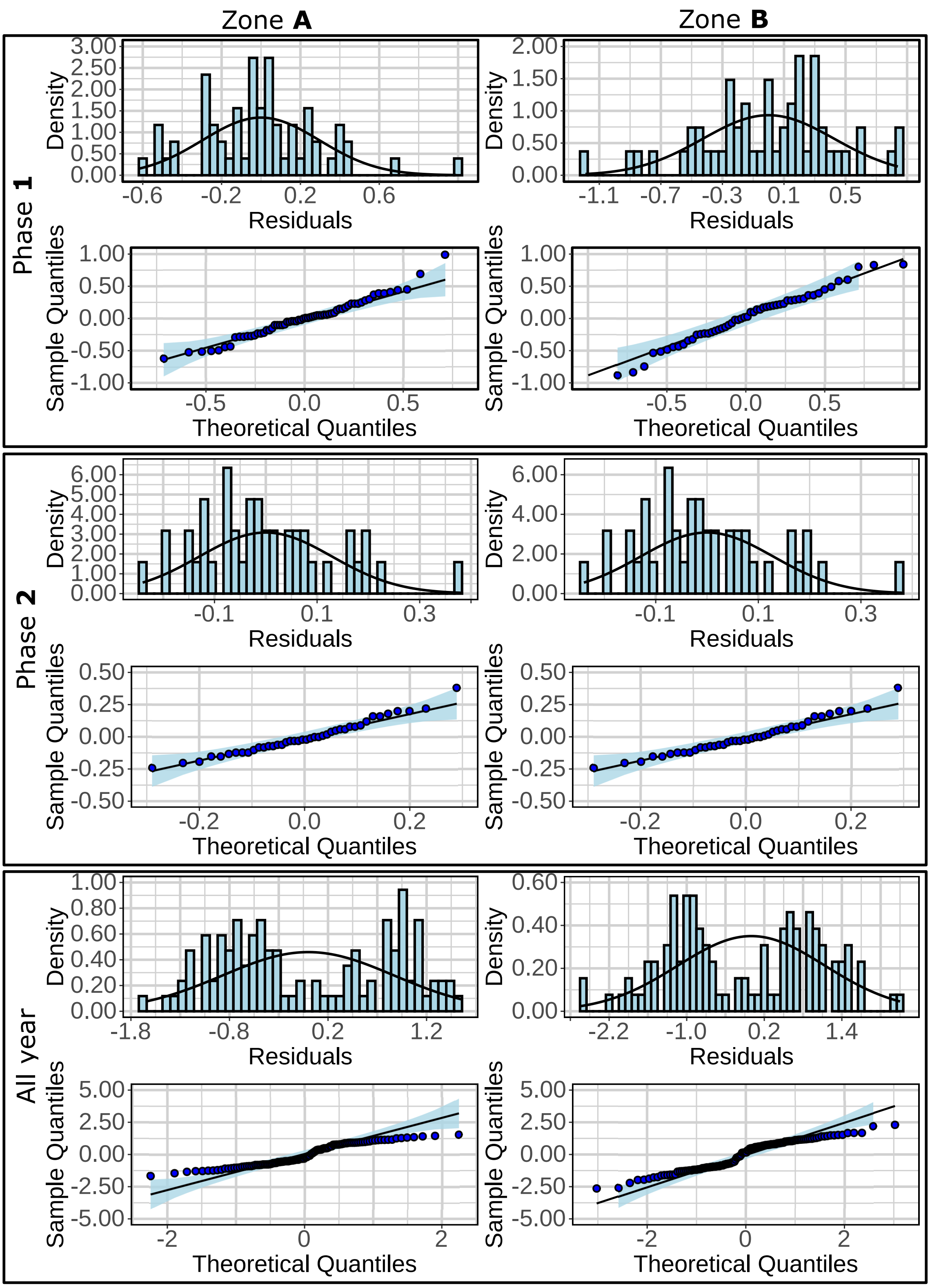

Figure S1. Histograms and fitted normal curves along with QQ plots of the residuals for linear regression for infections in Zone **A**, Zone **B** for Phase **1** (January, 2020 - June, 2020), Phase **2** (July, 2020 - December, 2020), and all year (2020).

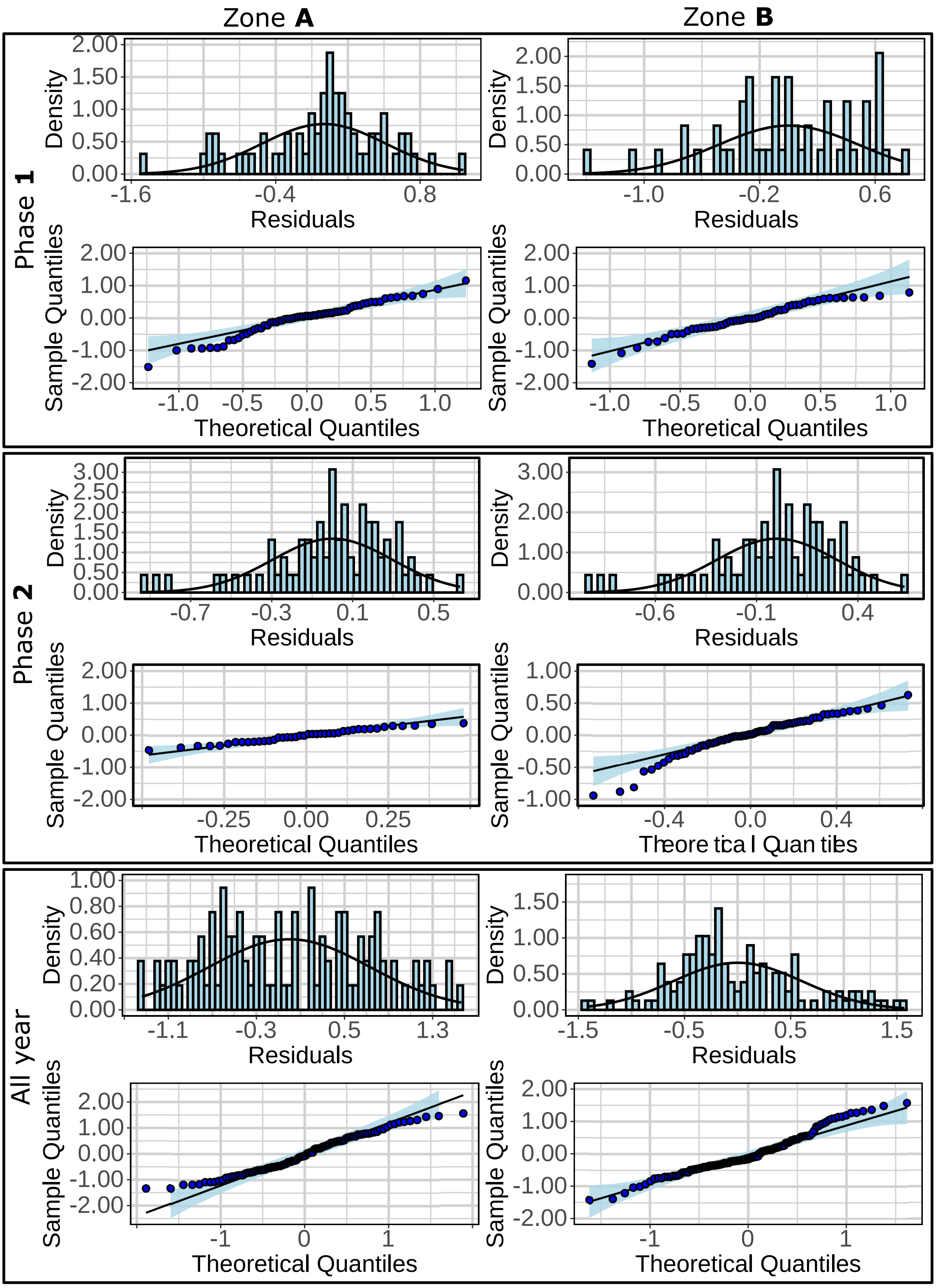

Figure S2. Histograms and fitted normal curves along with QQ plots of the residuals for linear regression for fatality in Zone **A**, Zone **B** for Phase **1** (January, 2020 - June, 2020), Phase **2** (July, 2020 - December, 2020), and all year (2020).

	Infection rate	Fatality rate	Population density	Age 65+	Poverty	African American	Hispanic American	NO_2	O_3	$PM_{2.5}$	SO_2	High school or less
Infection rate	1.00											
Fatality rate	0.18***	1.00										
Population density	0.41	0.14	1.00									
Age 65+	-0.12	0.53***	-0.34***	1.00								
Poverty	0.13	-0.24*	0.29**	-0.32***	1.00							
African American	-0.11	-0.16	0.23*	-0.25*	0.44***	1.00						
Hispanic American	0.33***	-0.26**	0.07	-0.20	0.24*	-0.37	1.00					
NO_2	0.08	0.16	0.41***	-0.26***	0.16	-0.01	0.22*	1.00				
O_3	-0.03	0.06	-0.15	0.13	-0.22*	-0.24	0.13	0.26**	1.00			
$PM_{2.5}$	-0.09	-0.10	0.12	-0.28	0.27**	0.30	0.12	0.35***	0.21	1.00		
SO_2	-0.12	0.19	0.07	0.01	0.28	0.13	-0.07	0.31**	0.05	0.44***	1.00	
High school or less	0.30**	0.12	-0.03	0.14	0.48***	-0.05	0.46***	0.09	0.10	0.22*	0.26**	1.00

Table S1. Pearson correlations between variables used in the study and their significance levels for Phase **1** Zone **A**.
'*' significance at $P < .10$, '**' significance at $P < .05$, '***' significance at $P < .01$

	Infection rate	Fatality rate	Population density	Age 65+	Poverty	African American	Hispanic American	NO_2	O_3	$PM_{2.5}$	SO_2	High school or less
Infection rate	1.00											
Fatality rate	0.05	1.00										
Population density	0.25*	-0.02	1.00									
Age 65+	-0.37**	0.35***	-0.06	1.00								
Poverty	0.11	-0.29**	-0.03	-0.25*	1.00							
African American	0.41***	0.01	0.36***	-0.20	0.27**	1.00						
Hispanic American	0.27**	-0.36***	0.05	-0.48***	0.33**	-0.23*	1.00					
NO_2	0.54***	0.05	0.20	-0.36***	0.15	0.16	0.38***	1.00				
O_3	0.24*	-0.06	-0.03	-0.20	0.25*	0.11	0.33**	0.52***	1.00			
$PM_{2.5}$	0.25*	-0.09	0.10	-0.26*	0.36***	0.25*	0.24*	0.37***	0.63***	1.00		
SO_2	-0.20	0.07	-0.05	0.40***	0.12	0.06	-0.20	0.10	0.14	0.08	1.00	
High school or less%	-0.03	-0.18	-0.28	0.22	0.34**	-0.04	0.23*	-0.09	0.31**	0.35**	0.29**	1.00

Table S2. Pearson correlations between variables used in the study and their significance levels for Phase **1** Zone **B**.
'*' significance at $P < .10$, '**' significance at $P < .05$, '***' significance at $P < .01$

	Infection rate	Fatality rate	Population density	Age 65+	Poverty	African American	Hispanic American	NO_2	O_3	$PM_{2.5}$	SO_2	High school or less
Infection rate	1.00											
Fatality rate	-0.10	1.00										
Population density	-0.10	-0.31**	1.00									
Age 65+	-0.24	0.39**	-0.14	1.00								
Poverty	0.03	0.31**	0.14	-0.13	1.00							
African American	-0.24	-0.06	0.42***	0.02	0.27*	1.00						
Hispanic American	0.27*	0.29*	-0.03	-0.36**	0.47***	-0.28	1.00					
NO_2	-0.05	0.06	0.16	-0.27*	0.22	0.06	0.40***	1.00				
O_3	-0.22	0.00	-0.16	-0.16	0.18	-0.27*	0.30*	0.53***	1.00			
$PM_{2.5}$	-0.11	-0.18	0.11	-0.22	0.34**	0.16	0.32**	0.27*	0.50***	1.00		
SO_2	-0.10	0.31**	-0.10	0.51***	0.11	0.00	-0.17	0.13	0.11	0.05	1.00	
High school or less%	0.00	0.46***	-0.09	0.03	0.61***	-0.05	0.57***	0.09	0.16	0.28*	0.22	1.00

Table S3. Pearson correlations between variables used in the study and their significance levels for Phase **2** Zone **A**.
'*' significance at $P < .10$, '**' significance at $P < .05$, '***' significance at $P < .01$

	Infection rate	Fatality rate	Population density	Age 65+	Poverty	African American	Hispanic American	NO_2	O_3	$PM_{2.5}$	SO_2	High school or less
Infection rate	1.00											
Fatality rate	0.06	1.00										
Population density	0.00	-0.13	1.00									
Age 65+	-0.18	0.38***	-0.37***	1.00								
Poverty	0.36***	0.19*	0.32***	-0.32***	1.00							
African American	0.26**	0.03	0.25**	-0.39***	0.48***	1.00						
Hispanic American	0.19*	-0.11	0.25**	-0.36***	0.08	-0.24**	1.00					
NO_2	0.22**	-0.17	0.48***	-0.35***	0.11	0.15	0.23**	1.00				
O_3	0.37***	-0.01	-0.01	0.03	-0.16	0.07	0.13	0.31***	1.00			
$PM_{2.5}$	0.43***	0.06	0.16	-0.25**	0.25**	0.35***	-0.04	0.37***	0.34***	1.00		
SO_2	0.21*	0.16	0.00	0.23**	0.18	0.06	-0.25**	0.06	0.04	0.35***	1.00	
High school or less%	0.29**	0.45***	0.06	0.33***	0.27**	0.06	0.07	-0.07	0.16	0.21*	0.27**	1.00

Table S4. Pearson correlations between variables used in the study and their significance levels for Phase **2** Zone **B**. '*' significance at $P < .10$, '**' significance at $P < .05$, '***' significance at $P < .01$

	Infection rate	Fatality rate	Population density	Age 65+	Poverty	African American	Hispanic American	NO_2	O_3	$PM_{2.5}$	SO_2	High school or less
Infection rate	1.00											
Fatality rate	-0.13	1.00										
Population density	-0.22	0.34**	1.00									
Age 65+	-0.31**	0.22	-0.25*	1.00								
Poverty	0.18	0.37**	0.17	-0.05	1.00							
African American	-0.23	0.09	0.24	-0.04	0.29*	1.00						
Hispanic American	0.37**	0.13	0.01	-0.28*	0.43***	-0.29*	1.00					
NO_2	-0.14	0.11	0.28*	-0.32**	0.14	0.03	0.36**	1.00				
O_3	-0.10	-0.15	-0.27*	-0.06	0.11	-0.25*	0.28*	0.44***	1.00			
$PM_{2.5}$	0.03	-0.27*	-0.04	-0.18	0.17	0.27*	0.22	0.24	0.44***	1.00		
SO_2	-0.16	0.16	-0.07	0.44***	0.11	0.01	-0.17	0.14	0.12	0.10	1.00	
High school or less%	0.22	0.27*	-0.17	0.07	0.57***	-0.02	0.50***	-0.07	0.20	0.27*	0.21	1.00

Table S5. Pearson correlations between variables used in the study and their significance levels for all year Zone **A**. '*' significance at $P < .10$, '**' significance at $P < .05$, '***' significance at $P < .01$

	Infection rate	Fatality rate	Population density	Age 65+	Poverty	African American	Hispanic American	NO_2	O_3	$PM_{2.5}$	SO_2	High school or less
Infection rate	1.00											
Fatality rate	0.05	1.00										
Population density	0.17	0.26**	1.00									
Age 65+	-0.25**	0.29**	-0.30**	1.00								
Poverty	0.30**	-0.15	0.28**	-0.35***	1.00							
African American	0.35***	-0.03	0.29**	-0.38***	0.46***	1.00						
Hispanic American	0.25**	-0.07	0.20*	-0.41***	0.13	-0.24**	1.00					
NO_2	0.25**	0.27**	0.46***	-0.30***	0.12	0.20*	0.19	1.00				
O_3	0.54***	0.14	0.06	-0.05	-0.10	0.05	0.18	0.35***	1.00			
$PM_{2.5}$	0.46***	-0.11	0.16	-0.29**	0.36***	0.26**	0.13	0.40***	0.42***	1.00		
SO_2	0.11	-0.03	0.01	0.27**	0.18	0.06	-0.25**	0.01	0.02	0.29**	1.00	
High school or less%	0.45***	0.29**	0.06	0.29**	0.31***	0.03	0.15	0.00	0.18	0.27**	0.29**	1.00

Table S6. Pearson correlations between variables used in the study and their significance levels for all year Zone **B**. '*' significance at $P < .10$, '**' significance at $P < .05$, '***' significance at $P < .01$

	Infection rate			Fatality rate		
**Variables**	**Coefficient**	***P*-value**	**VIF**	**Coefficient**	***P*-value**	**VIF**
Population density	0.00	0.000	1.46	0.00	0.002	1.46
Age 65+	-0.02	0.312	1.65	0.15	0.000	1.65
Poverty	-0.02	0.210	2.46	-0.05	0.045	2.46
African American	0.00	0.727	2.18	0.00	0.563	2.18
Hispanic American	0.00	0.911	2.15	-0.02	0.014	2.15
High school or less	0.03	0.005	2.12	0.04	0.008	2.12
NO_2	0.00	0.580	1.68	0.03	0.015	1.68
O_3	1.29	0.912	1.36	-17.88	0.379	1.36
$PM_{2.5}$	-0.05	0.172	1.77	-0.02	0.787	1.77
SO_2	-0.03	0.605	1.60	0.08	0.456	1.60
Sample size (n)	64			64		
Coefficient of determination (R^2)	0.37			0.57		

Table S7. Multiple linear regression on log-transformed response variables for Phase **1** Zone **A**
.

	Infection rate			Fatality rate		
**Variables**	**Coefficient**	***P*-value**	**VIF**	**Coefficient**	***P*-value**	**VIF**
Population density	0.00	0.873	1.56	0.00	0.310	1.56
Age 65+	-0.01	0.883	2.39	0.09	0.025	2.39
Poverty	-0.02	0.239	1.56	-0.01	0.617	1.56
African American	0.02	0.010	1.93	0.00	0.765	1.93
Hispanic American	0.00	0.485	2.75	-0.01	0.300	2.75
High school or less	0.01	0.703	2.20	-0.02	0.289	2.20
NO_2	0.04	0.005	1.94	0.03	0.046	1.94
O_3	1.76	0.930	2.19	-7.20	0.750	2.19
$PM_{2.5}$	0.02	0.711	2.14	0.02	0.698	2.13
SO_2	-0.09	0.006	1.49	-0.03	0.408	1.49
Sample size (n)	54			54		
Coefficient of determination (R^2)	0.53			0.32		

Table S8. Multiple linear regression on log-transformed response variables for Phase **1** Zone **B**
.

	Infection rate			Fatality rate		
**Variables**	**Coefficient**	***P*-value**	**VIF**	**Coefficient**	***P*-value**	**VIF**
Population density	0.00	0.526	1.34	0.00	0.053	1.34
Age 65+	-0.02	0.217	1.74	0.07	0.004	1.74
Poverty	-0.01	0.553	2.23	0.00	0.864	2.23
African American	0.00	0.209	2.32	0.01	0.134	2.32
Hispanic American	0.00	0.283	3.43	0.01	0.072	3.43
High school or less	-0.01	0.314	2.50	0.01	0.181	2.49
NO_2	0.00	0.934	2.41	0.00	0.615	2.41
O_3	-15.93	0.050	2.53	1.61	0.901	2.53
$PM_{2.5}$	0.01	0.771	1.88	-0.06	0.066	1.88
SO_2	0.01	0.448	1.90	0.01	0.794	1.90
Sample size (n)	42			42		
Coefficient of determination (R^2)	0.30			0.58		

Table S9. Multiple linear regression on log-transformed response variables for Phase **2** Zone **A**.

	Infection rate			Fatality rate		
**Variables**	**Coefficient**	***P*-value**	**VIF**	**Coefficient**	***P*-value**	**VIF**
Population density	0.00	0.077	1.57	0.00	0.471	1.57
Age 65+	-0.01	0.544	2.63	0.05	0.031	2.63
Poverty	0.01	0.147	1.81	0.02	0.129	1.81
African American	0.00	0.217	2.08	0.00	0.451	2.08
Hispanic American	0.01	0.074	1.93	0.00	0.483	1.93
High school or less	0.01	0.238	1.74	0.01	0.075	1.74
NO_2	0.00	0.578	1.72	0.00	0.828	1.72
O_3	28.29	0.003	1.47	-7.32	0.494	1.47
$PM_{2.5}$	0.04	0.189	1.80	0.02	0.542	1.80
SO_2	0.05	0.174	1.42	-0.02	0.674	1.42
Sample size (n)	76			76		
Coefficient of determination (R^2)	0.47			0.29		

Table S10. Multiple linear regression on log-transformed response variables for Phase **2** Zone **B**.

	Infection rate			Fatality rate		
**Variables**	**Coefficient**	***P*-value**	**VIF**	**Coefficient**	***P*-value**	**VIF**
Population density	0.00	0.023	1.51	0.00	0.097	1.51
Age 65+	-0.03	0.017	1.64	0.06	0.015	1.64
Poverty	0.01	0.154	2.28	0.01	0.684	2.27
African American	0.00	0.126	2.36	0.00	0.581	2.36
Hispanic American	0.00	0.372	3.20	0.00	0.900	3.20
High school or less	0.00	0.741	2.50	0.02	0.046	2.50
NO_2	0.00	0.440	2.63	0.02	0.090	2.63
O_3	-12.79	0.067	2.38	-12.84	0.361	2.38
$PM_{2.5}$	0.02	0.364	1.90	-0.06	0.109	1.90
SO_2	0.00	0.749	1.89	-0.01	0.763	1.89
Sample size (n)	45			45		
Coefficient of determination (R^2)	0.45			0.48		

Table S11. Multiple linear regression on log-transformed response variables for all year Zone **A**.

	Infection rate			Fatality rate		
**Variables**	**Coefficient**	***P*-value**	**VIF**	**Coefficient**	***P*-value**	**VIF**
Population density	0.00	0.673	1.47	0.00	0.033	1.47
Age 65+	-0.02	0.149	2.83	0.05	0.083	2.83
Poverty	-0.01	0.470	1.96	-0.02	0.230	1.96
African American	0.01	0.013	2.06	0.00	0.777	2.06
Hispanic American	0.00	0.244	2.14	-0.01	0.270	2.14
High school or less	0.02	0.005	1.85	0.03	0.005	1.85
NO_2	0.00	0.953	1.64	0.02	0.009	1.64
O_3	37.42	0.000	1.52	4.92	0.719	1.52
$PM_{2.5}$	0.01	0.643	1.95	-0.07	0.116	1.95
SO_2	0.03	0.274	1.43	-0.06	0.267	1.43
Sample size (n)	73			73		
Coefficient of determination (R^2)	0.60			0.43		

Table S12. Multiple linear regression on log-transformed response variables for all year Zone **B**.

	$PM_{2.5}$					
	Phase **1**		Phase **2**		All year	
	Zone **A**	Zone **B**	Zone **A**	Zone **B**	Zone **A**	Zone **B**
ARIMA	0.88	1.14	1.08	0.88	1.06	0.90
Exponential smoothing	0.86	0.86	0.88	0.81	0.83	0.84
Autoregressive neural network	0.91	0.81	0.96	0.81	0.93	0.81
	NO_2					
	Phase **1**		Phase **2**		All year	
	Zone **A**	Zone **B**	Zone **A**	Zone **B**	Zone **A**	Zone **B**
ARIMA	0.64	0.63	0.59	0.61	0.62	0.59
Exponential smoothing	0.59	0.61	0.55	0.58	0.60	0.61
Autoregressive neural network	0.71	0.68	0.32	0.69	0.68	0.70
	SO_2					
	Phase **1**		Phase **2**		All year	
	Zone **A**	Zone **B**	Zone **A**	Zone **B**	Zone **A**	Zone **B**
ARIMA	0.06	0.08	0.10	0.10	0.07	0.10
Exponential smoothing	0.07	0.08	0.10	0.10	0.08	0.10
Autoregressive neural network	0.05	0.06	0.07	0.06	0.05	0.06

Table S13. The root mean squared error (RMSE) values to show of the the accuracy of forecasting models' predicted values versus the actual or observed values (RMSE values of $O_3 < 0.001$ for Phase **1**, Phase **2** and all year), Abbreviation: ARIMA- autoregressive integrated moving average.
